# Insights From the Development of a Dynamic Consent Platform for the Australians Together Health Initiative (ATHENA) Program: Interview and Survey Study

**DOI:** 10.2196/57165

**Published:** 2024-11-06

**Authors:** Eddy Xiong, Carissa Bonner, Amanda King, Zoltan Maxwell Bourne, Mark Morgan, Ximena Tolosa, Tony Stanton, Kim Greaves

**Affiliations:** 1 School of Medicine and Dentistry Griffith University Southport, Queensland Australia; 2 The Australians Together Health Initiative Program (The ATHENA Program) Sunshine Coast University Hospital Queensland Health Birtinya, Queensland Australia; 3 Menzies Centre for Health Policy and Economics School of Public Health, Faculty of Medicine and Health University of Sydney Camperdown, New South Wales Australia; 4 Maleny Doctors Maleny, Queensland Australia; 5 Faculty of Health Sciences and Medicine Bond University Gold Coast, Queensland Australia; 6 Communicable Disease Branch Queensland Department of Health Herston, Queensland Australia; 7 Department of Cardiology Sunshine Coast University Hospital Queensland Health Birtinya, Queensland Australia; 8 National Centre for Epidemiology and Population Health ANU College of Health and Medicine Australian National University Canberra, Australian Capital Territory Australia

**Keywords:** dynamic consent, research, clinical trials, consumer engagement, digital consent, development, decision making, decision, feedback, user platform, users, communication

## Abstract

**Background:**

Dynamic consent has the potential to address many of the issues facing traditional paper-based or electronic consent, including enrolling informed and engaged participants in the decision-making process. The Australians Together Health Initiative (ATHENA) program aims to connect participants across Queensland, Australia, with new research opportunities. At its core is dynamic consent, an interactive and participant-centric digital platform that enables users to view ongoing research activities, update consent preferences, and have ongoing engagement with researchers.

**Objective:**

This study aimed to describe the development of the ATHENA dynamic consent platform within the framework of the ATHENA program, including how the platform was designed, its utilization by participants, and the insights gained.

**Methods:**

One-on-one interviews were undertaken with consumers, followed by a workshop with health care staff to gain insights into the dynamic consent concept. Five problem statements were developed, and solutions were posed, from which a dynamic consent platform was constructed, tested, and used for implementation in a clinical trial. Potential users were randomly recruited from a pre-existing pool of 615 participants in the ATHENA program. Feedback on user platform experience was gained from a survey hosted on the platform.

**Results:**

In the 13 consumer interviews undertaken, participants were positive about dynamic consent, valuing privacy, ease of use, and adequate communication. Motivators for registration were feedback on data usage and its broader community benefits. Problem statements were security, trust and governance, ease of use, communication, control, and need for a scalable platform. Using the newly constructed dynamic consent platform, 99 potential participants were selected, of whom 67 (68%) were successfully recontacted. Of these, 59 (88%) agreed to be sent the platform, 44 (74%) logged on (indicating use), and 22 (57%) registered for the clinical trial. Survey feedback was favorable, with an average positive rating of 78% across all questions, reflecting satisfaction with the clarity, brevity, and flexibility of the platform. Barriers to implementation included technological and health literacy.

**Conclusions:**

This study describes the successful development and testing of a dynamic consent platform that was well-accepted, with users recognizing its advantages over traditional methods of consent regarding flexibility, ease of communication, and participant satisfaction. This information may be useful to other researchers who plan to use dynamic consent in health care research.

## Introduction

Rapid advances in medical knowledge, clinical trials, and research have necessitated new and innovative requirements for obtaining informed consent. Dynamic consent is a proposed solution and is enabled by concurrent developments in technology [1,2].

Dynamic consent is a consent framework that encourages research participants to take a more active role when consenting to take part in research studies and clinical trials [3]. For example, participants can update their preferences in real time, and this can be managed through an online platform [4]. In doing so, studies can more effectively meet the legal and ethical standards that are ever-changing in the modern research environment and require a more flexible approach to ensure safety and autonomy [5]. The increasingly complex nature of studies, such as those in the fields of genomics and epidemiology, has given rise to much broader and more lengthy consent processes, which have made it progressively more difficult for participants to understand the terms involved [6]. This results in a system that fails to adequately address participant understanding and comprehension [7,8]. Insufficient understanding of concepts in clinical trials is common due to the quantity and complexity of the information and can result in withdrawal of participation [7,9]. This has contributed to the great “connection” issue affecting clinical trials regarding participant recruitment, retention, and engagement [10]. This reflects a failure on the part of the researchers to meet their needs by minimizing participant burden, building trust, and ensuring comprehension of study goals and risks [11,12].

Since its initial proposal over a decade ago, dynamic consent has remained a topic of ongoing debate, and implementations are being trialed to build an evidence base to inform use [1,13,14]. Nevertheless, dynamic consent holds the promise of addressing many of the issues mentioned above. Operating on a web-based platform, the potential exists for participants to have higher levels of data control, be provided with more information about outcomes and data reuse, and have more interactive and engaging mediums of information delivery [15,16]. Being able to consent digitally has shown demonstrable benefits in engagement and comprehension of content compared with traditional consent processes [7]. Other features include the ability to provide and retract consent at will and in real time, and provide a direct and ongoing communication link between participants and researchers. Due to its versatility, dynamic consent has been used in the generation of large-scale biomedical databases (biobanks) for research, containing the health information, including biospecimens, of several hundred thousand participants [16-18]. Past methods have traditionally relied on broad or blanket consent, a method that risks infringing upon participant autonomy, requiring careful consideration to meet ethical and legal requirements [19,20]. Dynamic consent potentially mitigates this by offering the possibility of consent choices, allowing specification of which information can be used for research [13]. Participants can also receive feedback on when, why, and who has accessed their health information, and they are able to withdraw consent or update their consent preferences at any time. Other applications include the ability to prospectively consent to be contacted by researchers in the future and having control of the frequency of communications with researchers. These features mean that participants can be more engaged in the decision-making process and aware of the broader significance of their contribution to the project, thus theoretically fostering greater satisfaction and retention when taking part in clinical trials [4,16,21].

The Australians Together Health Initiative (ATHENA) program was conceptualized as a state-wide program for Queensland, Australia, with the central vision of engaging and connecting researchers and trialists with patients across the state, thus providing them with a comprehensive picture of ongoing research activities, new treatment options, and opportunities to participate in research [22,23]. At the core of this approach is dynamic consent, which aims to systematically engage and recruit over 1 million people attending the health system. ATHENA focuses on obtaining consent on 2 crucial fronts. First, participants are invited to share access to their health information with the state government health department. This includes data from primary care providers, hospitals, and health registries, as well as any routinely collected administrative health registry information, for research purposes. Second, ATHENA actively seeks consent to allow for the recontact of participants, leading to the formation of a substantial cohort of research-willing participants. The resultant health data collection can be screened to rapidly identify and contact eligible participants for clinical trials or research studies, thus streamlining the whole trial design and recruitment process. This has the potential to solve the significant recruitment challenges that currently plague clinical research both nationally and internationally [11]. The benefits of the ATHENA program for patients include access to additional treatment options, a more active role in research, improved population health, access to personalized mutually beneficial research opportunities, and long-term engagement with the research community. For researchers, the program offers an enhanced study design, efficient and rapid participant recruitment, reduced risk and cost in recruitment, simplified research processes, and expanded research exposure, and facilitates beneficial inclusions within grant applications. In 2020, ATHENA successfully undertook the ATHENA COVID-19 feasibility study aimed at generating a cohort of patients diagnosed with COVID-19 in Queensland who had consented to provide a comprehensive set of their clinical information, including linked general practice, hospital, and other registry health information, as well as consented for recontact [22,23]. The purpose of the study was to provide a cohort of patients who could be recontacted for future COVID-19–related research, test the ATHENA concept, and gain some understanding of the epidemiology of patients with COVID-19.

Although relatively new, several other groups globally have successfully integrated a dynamic consent platform into their existing research infrastructure. The consent platform developed for ATHENA possesses several features that distinguish it from other platforms. Unlike its counterparts [16,17,24,25], the ATHENA dynamic consent platform has been developed with the future capability for broad-scale, mass recruitment in mind, rather than being disease- or trial-specific. It also takes a relatively systematic rather than opportunistic approach to participation, with registration being offered as part of routine hospital care. Another difference is that it requests linkage of health information for unspecified research in the future and introduces consent to recontact.

This report aims to describe the development of the ATHENA dynamic consent platform within the framework of the ATHENA program [22], including how the platform was designed, its utilization by participants, and the insights gained. This information may be useful to those who wish to develop a dynamic consent platform for future medical research.

## Methods

### Overview

**Figure 1:**
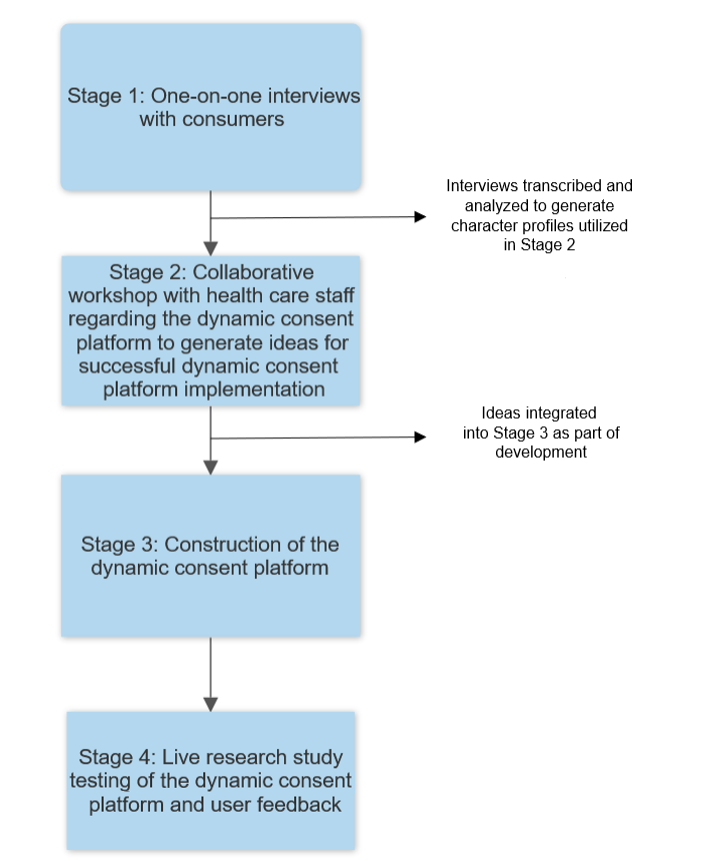
Flowchart of the development and testing process for the Australians Together Health Initiative (ATHENA) dynamic consent platform.

The development and testing approach of the ATHENA concept and dynamic consent platform was divided into 4 stages (Figure 1): (1) one-on-one interviews with consumers regarding dynamic consent in the context of the ATHENA program; (2) a collaborative workshop with health care staff for insights regarding the dynamic consent platform; (3) construction of the dynamic consent platform; and (4) live research study testing of the dynamic consent platform and user feedback.

### Stage 1: One-on-One Interviews With Consumers Regarding the Concept of Dynamic Consent in the Context of the ATHENA Program

The first stage of development was outsourced to the Digital Innovation Hub within Queensland Health, which undertook one-on-one interviews with consumers, with specific consideration given to consumer opinions on the concept of dynamic consent within the ATHENA program. Participants were recruited from an established pool of volunteers and through advertisements run at the local hospital and health service. Participants were selected to represent a broad range of demographics: (1) participants from culturally and linguistically diverse, indigenous, and Caucasian communities; (2) participants aged 21-74 years; (3) participants living in rural and urban communities; and (4) participants having a variety of professions, including manual workers, carers, IT specialists, and general practitioners.

Each individual underwent a semistructured interview for 1 hour, which was assisted with a discussion guide centered around several key areas: health and technology literacy, understanding of data privacy and trust in security measures, the concept of dynamic consent and data sharing, motivation to sign up for the ATHENA program, and barriers to recontact.

A summary of the discussion guide is provided in Multimedia Appendix 1 [26]. Interviews were conducted either in-person or through an online video call and were recorded. Interviews were transcribed, and thematic analysis was performed to extract several broad themes representative of the beliefs held by participants. These themes were used to generate several different character profiles, which demonstrated the differing opinions regarding dynamic consent, the ATHENA program, and the broader themes identified. Character profiles were additionally assigned demographics such as occupation, residence, and age. These represented an amalgamation of interviewees’ beliefs and did not represent any specific individual, and they played a role in the second stage of dynamic consent platform construction by providing examples of potential types of users for consideration.

### Stage 2: Collaborative Workshop With Health Care Staff Regarding the Dynamic Consent Platform

This stage involved a 1-day workshop involving guided group discussions with key stakeholders from health care. The character profiles from stage 1 were presented to the group, and several prompts and activities were enacted to encourage discussion and consideration of various aspects of the dynamic consent model. Key discussion points centered around roadblocks hindering access to the dynamic consent platform for users, as well as any potential solutions. Participants were encouraged to generate a set of “problem statements” that would be necessary constituents of a successful dynamic consent platform and were then asked to propose potential solutions or features that would address these statements. The knowledge gained from this workshop was then integrated into the design of the dynamic consent platform.

### Stage 3: Construction of the Dynamic Consent Platform

Using the information gained from stages 1 and 2, the ATHENA dynamic consent platform was constructed by eHealth Queensland. To meet local ethics requirements, the use of the dynamic consent platform required compliance with Queensland Health’s legal and cybersecurity standards. Consultations with both Queensland Health cybersecurity and legal teams were undertaken.

### Stage 4: Live Research Study Testing of the Dynamic Consent Platform and User Feedback

The cohort of the ATHENA COVID-19 study consisted of 995 participants who had been diagnosed with COVID-19, of whom 842 (84.6%) were successfully contacted and reached a consent decision regarding recontact. In this cohort, 615 (73.0%) consented to future recontact to discuss participation in COVID-19–related research trials or dynamic consent participation [22]. Due to resource constraints, some of these individuals (n=99) were randomly selected to be recontacted to consent for testing the dynamic consent platform. Each participant was contacted via telephone to ask if they would agree to receive information about a new research study called the COVID OZ-Genetics Research Project [27]. Those who expressed interest and consented were registered on the dynamic consent platform and emailed a login link with a password. The web link to the platform was then emailed separately to participants asking them to log in using the details provided. Upon signing into the platform, participants were presented with a summary of the new clinical trial opportunity: the COVID OZ-Genetics Research Project. Interested participants were prompted to go through a short series of steps on the platform requesting consent to have their contact details released to the COVID OZ-Genetics trial team, who would then contact them to discuss the study in more detail. The COVID OZ-Genetics Research Project is a study run by the University of Queensland, investigating genetic factors that may predispose individuals to long COVID [27]. While on the dynamic consent platform, participants were also invited to complete a 5-point Likert survey that captured feedback on their experience using the dynamic consent platform, which is a modified version of a published survey [26]. Response options available were “strongly agree” (score 1), “agree” (score 2), “neither agree nor disagree” (score 3), “disagree” (score 4), and “strongly disagree” (score 5). The questionnaire and answer key can be viewed in Multimedia Appendix 2. The CHERRIES (Checklist for Reporting Results of Internet E-Surveys) was also completed and can be viewed in Multimedia Appendix 3 [28].

### Ethical Considerations

Ethics approval for this study was granted by the Gold Coast Hospital and Health Service Human Research Ethics Committee (reference: HREC/2020/QGC/63555) and the Australian National University Human Research Ethics Committee (reference: 2020/312).

## Results

### Stage 1: One-on-One Interviews With Consumers Regarding the Concept of Dynamic Consent in the Context of the ATHENA Program

Thirteen one-on-one interviews were conducted, and participant demographics are summarized in Table 1.

**Table 1:** Demographics of participants interviewed for the development of the Australians Together Health Initiative (ATHENA) dynamic consent platform.

Demographics		Value (N=13), n (%)
**Gender**		
	Male	6 (46)
	Female	7 (54)
**Age (years)**		
	18-24	1 (9)
	25-34	2 (15)
	35-44	2 (15)
	45-54	2 (15)
	55-64	2 (15)
	65-74	4 (31)
**Residential location**		
	Urban	9 (69)
	Rural	4 (31)
	Identifies as Aboriginal and Torres Strait Islander (yes)	1 (9)
**Relationship with technology**		
	“It helps me a lot in my life, but I don’t need it all the time”	2 (15)
	“I use it mostly for work”	2 (15)
	“I use it for a range of things, but it doesn’t feature much in my life”	2 (15)
	“I love it all”	5 (39)
	“I hardly ever use digital technology, but I know it’s there if I ever need it”	2 (15)

Four themes were identified through thematic analysis of interviews. The first theme was consent and control. Participants identified that it would be important for them to be able to play a role and have control in the sharing of identifiable data. They were supportive of a digital platform that would be an effective means of enabling them to update their preferences in real time, so long as assurances were made regarding data security. The second theme was motivation to register, benefit to the community, and feedback on outcomes. Multiple participants noted that financial incentives would not be an effective motivator for registration. The notion of receiving feedback and updates regarding the outcomes of their participation in the study was met with enthusiasm, and participants expressed that this would be a good motivating factor to participate. Several participants observed that this was a point of dissatisfaction with previous trials they had participated in. They stated that after their involvement finished, they were never informed about the outcome or results of the trial. It was also noted that if it could be demonstrated that their participation was of benefit to the health of the wider community, this would likely be the greatest motivator for registration. Interviewees also identified that easy access and registration on the platform were important. The majority also stated that a recommendation from their general practitioner to register with ATHENA would play a significant role in their decision to sign up. The third theme was data privacy and communication. Participants identified both the benefits that sharing their data would bring and the potential risk for data breaches. The desire for assurance that their data would be safely stored was a common theme. There was a relatively high level of trust placed in the government and Queensland Health to maintain data security. Participants also had strong opinions on their preferences for the frequency of contact from researchers, identifying that this should be something that can be personalized during the registration process. The fourth theme was dynamic consent. Participants were universally positive about the concept of dynamic consent. They demonstrated an understanding and appreciation of its benefits and how this would allow for many of the conditions outlined above to be met. Many expressed the opinion that participant consent should be acquired whenever the sharing of information is involved, and acknowledged that a dynamic consent platform, if operated correctly, would be an effective means to accomplish this.

Six character “profiles” were generated, which are presented in Table 2, representing both the common themes and beliefs identified through thematic analysis, as well as any potential fringe beliefs and ideas regarding dynamic consent identified in the interviews conducted.

**Table 2:** Summary of amalgamated character profiles generated from one-on-one interviews conducted for the Australians Together Health Initiative (ATHENA) program, each expressing differing opinions regarding dynamic consent.

Background (age in years, occupation, and location)	Security/trust	Sharing of data	Communication	Summative quote
A 50-year-old tradesman who lives in a rural area	Open minded about data sharing.	Would sign up for the platform if they saw the benefits of the platform.	Minimal contact preferred. Emails only.	“Data can be good, like when I had a work safety compensation case, it helped me get the facts and get sorted.”
A 21-year-old IT worker who lives near the city	Does not want data being shared unless it is relevant for their health.	Happy to share data if it is anonymous.	Would wait to see demonstrable benefits before registering.	“If I can’t do it online with the data, then I won’t do it at all. I spend most of my time connected to something.”
A 43-year-old general manager of a technology company who lives in the city	Expresses concerns regarding security and transparency around personal data.	Identified that there are many positives with sharing data. Would be annoyed if data were shared without consent.	Would like to have communication regarding what data are being shared.	“I need to know the governance and protective measures for my data.”
A 70-year-old retired person who lives in a semiurban area	Has “nothing to hide” and is not concerned about data privacy.	“If the data can help someone else, then just do it.”	Would like to know how their data are used. “It is important to close the loop.”	“I don’t worry about privacy when it comes to my information. I think sharing of this data is very necessary and I would just let it happen.”
A 32-year-old product developer who lives in the city	Trusts the government to store and share data securely.	Happy to share data as long as it is not identifiable.	Would like to be consented for absolutely every study.	“Data is a powerful tool and can be used to better or worsen the world.”
A 40-year-old unpaid volunteer who lives in community housing	Trusts the government with their data. “If you can’t trust them, who can you trust?”	Happy to share data as long as it cannot be used to discriminate against them.	Would use their general practitioner as their point of communication. Great deal of trust in their general practitioner.	“I am happy to share my data to help someone else, but I want it anonymized.”

### Stage 2: Collaborative Workshop With Health Care Staff Regarding the Dynamic Consent Platform

Six health care staff were involved in the workshop and included a medical consultant, a general practitioner, an IT specialist, a clinical trials nurse specialist, a clinical nurse, and a senior manager representing clinical trials and research for Queensland Health. Several factors were identified that would be important in ensuring the successful uptake of dynamic consent. From these, 5 central “problem statements” (listed below) were generated by workshop participants to aid further discussion and development. The first statement involved security, trust, and governance. Users should be able to trust that their information is safe, secure, and appropriately governed, so that they can be confident their information will only be used by the right people, for the intended purpose. The second statement involved ease of use. Users should be able to intuitively navigate the platform and get simple, clear guidance and information on the research trials available. This enables them to be better informed when making decisions about the platform. Additionally, a specific issue identified was the lengthy nature of participant information consent forms, compounded by insufficient health literacy. Stakeholders identified that patients often feel overwhelmed and confused by the inconsistency of formats presented in consent forms, the burden of form filling, and the difficulty in finding a suitable study to participate in. The third statement involved control. Users should be able to easily sign up to the platform on any device, manage their preferences, stay engaged with the platform, and be able to opt-out so that they have full control of their information. The fourth statement involved ease of communication. Users should be able to easily communicate with researchers and staff to take part in research trials or provide feedback or progress on research outcomes, saving time on recruitment and maintaining engagement. The fifth statement involved a scalable system. Queensland Health staff and researchers should have access to a scalable, adaptable system, which can be used for other types of consent such as for health procedures.

Workshop participants were then asked to propose solutions to the problem statements, which are summarized in Table 3.

**Table 3:** Solutions proposed by health care staff to manage problem statements arising from the Australians Together Health Initiative (ATHENA) dynamic consent workshop, and their incorporation into the new platform build.

Problem statement, section of the platform, solution			Incorporation into the final platform build (Yes/No)
**Ease of use; Ease of communication**			
	**General platform features**		
		Must be a web application or an app that is intuitive and self-explanatory	Yes
		Ability to send text messages or emails to participants	Yes (email)
**Security, trust, and governance; Ease of communication**			
	**Login**		
		Secure authentication system	Yes
		Frequently Asked Questions section (for instance, regarding data usage)	Yes
		Progress bar for sign-up (estimation of time remaining)	No
**Ease of use; Control**			
	**About you (personal page)**		
		How many trials the participant is already involved in	Yes
		What research papers their data have contributed to	No
		Virtual medals or other rewards for incentivizing and rewarding participation	No
		Easy withdrawal section or opt-out	Yes
**Security, trust, and governance**			
	**Who the ATHENA** ^a^ **program is supported by**		
		General practitioners	Yes
		Royal Australian College of General Practitioners	Yes
		Primary health networks	Yes
		Queensland Health	Yes
		Heart Foundation	No
		Cancer research	No
**Ease of use**			
	**Participant information sheet and consent form**		
		As simplified as possible	Yes
		Use drop-down menus that reveal lists of options or sections	Yes
**Ease of use**			
	**What’s new section**		
		Outline of what new trials are available	Yes
		Ability for the patient to search for a trial related to a specific condition	No
**Ease of communication**			
	**Contact us section**		
		Refer a friend or relative	No
		Contact details	Yes
		Help desk	Yes
		Chatbot	No
**Security, trust, and governance; Scalable system**			
	**Backend**		
		Audit trail	Yes
		Participant profiling	No
		Automated release of data from the general practitioner straight to Queensland Health upon consent	No
**Ease of use; Ease of communication**			
	**Information or landing page**		
		Introductory video	No
		Description of the dynamic consent platform/ATHENA	Yes
		Frequently Asked Questions section	Yes
		Strong purpose statement – How will the participant’s involvement help?	Yes
**Ease of communication**			
	**Help and support**		
		Chat box that connects the participant to a staff member	No
**Ease of use**			
	**Continued engagement**		
		Badges or rewards for participation	No
**Security, trust, and governance; Control; Scalable system**			
	**Other considerations**		
		End-user testing	Yes
		Death data linkage so participants who have passed away are not contacted mistakenly or so their next of kin may be contacted	No
		Legal and ethics review	Yes

^a^ATHENA: Australians Together Health Initiative.

### Stage 3: Construction of the Dynamic Consent Platform

#### Architectural IT Concept Design

An architectural IT concept design (Multimedia Appendix 4) for the dynamic consent platform was drawn up by eHealth Queensland containing the core requirements and solutions identified from stages 1 and 2. It was the intention to implement most of these features in the pilot platform trialed in this study. Some were not implemented due to time and cost restrictions, and these would be implemented in a later version of the platform (Table 3).

#### Dynamic Consent Platform Construction

The dynamic consent platform was built using a health care intelligence solutions platform already in use by the Queensland public health system to ensure scalability. This platform enables the acquisition and analysis of performance analytics, data visualization, and user experience analytics. This is crucial to enable the project to better understand user engagement, demographics, retention, and behavior. All data were stored in Queensland Health, and all data changes were audited and can be viewed via comprehensive audit logs.

There are 3 different user groups that will use the platform: general users (participants), platform reporting or dashboard users, and administrator users. General users can create new consent choices and provide input for surveys. Reporting or dashboard users are responsible for the day-to-day operations of the platform and can create new dashboards and run reports. Administrators have full access to create new content, such as records and study templates, and are responsible for updating content as required (for example, for newly available clinical trials or changes to existing trials). They can review actions made by participants, such as study project choices, emails requested, and survey submissions. They can edit some features of the website, such as menu and list views. Importantly, no users have the capacity to edit consent responses.

**Figure 2:**
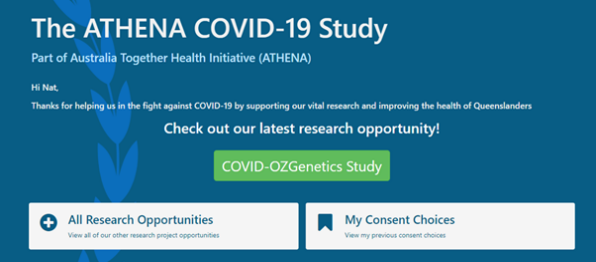
Image of the landing page for the ATHENA dynamic consent platform webpage. ATHENA: Australians Together Health Initiative.

**Figure 3:**
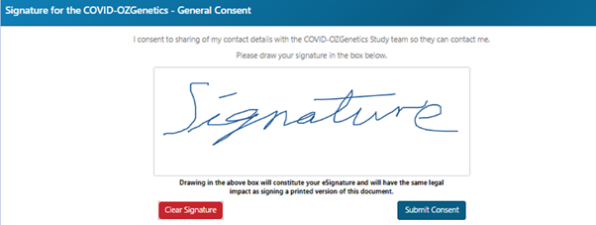
Image of a sample digital signature consent form on the Australians Together Health Initiative (ATHENA) dynamic consent platform webpage.

The platform was designed with clarity and accessibility in mind. Initially, users are brought to an information page with an introduction and frequently asked questions section. After registration, a landing page for the dynamic consent platform is presented (Figure 2), from which studies available to participate are visible. Upon selecting a study, the user is presented with the title and details of the study. Consent is submitted via a digital signature (Figure 3), and participants are provided a digital receipt, which can be viewed within the dynamic consent platform. They will also be sent a confirmation email with a PDF copy of their submitted consent. Participants can withdraw consent. A full set of images demonstrating this registration process can be seen in Multimedia Appendix 5.

#### Legal and Cybersecurity Compliance

Advice from Queensland Health’s legal and cybersecurity teams has been incorporated into the build to ensure that the platform meets the required standards. Legal requirements necessitated compliance with multiple “Health Acts” and the completion of a “Privacy Impact Assessment” [29-34]. Cybersecurity required that safety standards were complied with regarding the collection, storage, and use of participant information; that appropriate levels of administrative and technical security provisions were adopted to protect the personal information stored in or used by the platform; and that Australian Privacy Principle 4 of the Australian Privacy Principles Act was met [31].

### Stage 4: Live Research Study Testing of the Dynamic Consent Platform and User Feedback

A total of 99 participants were identified, of whom 67 (68%) were successfully recontacted. Of these, 59 (88%) agreed to be sent the dynamic consent platform and 8 (12%) declined. A total of 44 (75%) logged onto the platform (indicating use), and 25 (57%) of these expressed interest in the new research study and gave consent for their contact details to be released to the OZ-Genetics study team. No participants actively refused consent. Eventually, 17 (68%) participants completed the posttrial survey regarding experiences with the dynamic consent platform and general feedback. Multimedia Appendix 6 summarizes the flow of participant recruitment. Table 4 summarizes the participant response rates of the recruitment process. For simplicity, responses (1) and (2) are grouped into “agree,” response (3) is “neutral,” and responses (4) and (5) are grouped into “disagree.”

**Table 4:** Survey responses regarding the usability of the Australians Together Health Initiative (ATHENA) dynamic consent platform collected after a trial of the platform.

Question	Response (N=17), n (%)		
	Agree	Disagree	Neutral
My overall experience using this website was good.	13 (76)	2 (12)	2 (12)
It was easy to navigate the website.	13 (76)	2 (12)	2 (12)
The length of time it took me to complete my consent choice was too long.	3 (18)	12 (71)	2 (12)
The information presented to me in the consent choice process was clear.	14 (82)	2 (12)	1 (6)
The amount of information provided to allow me to make a consent choice was just right.	15 (88)	2 (12)	0 (0)
I felt pressured to complete the consent process.	1 (6)	16 (94)	0 (0)
I liked the opportunity to be able to provide consent choices.	12 (71)	1 (6)	4 (24)
The number of consent choices provided to me was just right.	13 (76)	1 (6)	3 (18)
I felt positively about the opportunity to ask questions and provide feedback to the research team.	12 (71)	1 (6)	4 (24)

## Discussion

### Key Findings

This study describes the development of a dynamic consent platform within the framework of the ATHENA program. A multi-faceted research methodology was used to explore the acceptability of the program, including the processes involved in the development and testing of a dynamic consent platform, one-on-one consumer interviews, workshops with health care staff, construction of a dynamic consent platform, and testing within an active research study. Key findings from the perspective of participants were control of data, feedback, communication, and data privacy. It also reflected that participants were broadly receptive to the concept of dynamic consent if implemented appropriately. Stage 2 translated this information into a set of problem statements with practical solutions, which could be directly incorporated into the construction of the platform. Following construction of the platform (stage 3), stage 4 demonstrated that by implementing these features, we were successful in creating a platform whereby 75% of those participants who agreed to be sent the platform logged on (indicating use). In addition, the platform received positive feedback from users who completed the survey.

### The Role of Dynamic Consent and Comparison With Prior Research

Although the ATHENA dynamic consent platform concept received positive acceptance by the public, this was subject to certain conditions. These included having control over data, use of data for public benefit, feedback on the use of health information, and ensuring privacy, especially where identifiable data were used. The use of dynamic consent was widely accepted as a means to achieve this, with participants cognizant of its potential advantages over paper-based or simple electronic-based consent, and how its use might meet the many conditions described above. Our methodology identified that participants have a strong preference for being able to exercise control of the distribution of personal data, which is consistent with the literature [6,16]. As dynamic consent is only as effective as users’ willingness to engage with the platform, it is imperative that such needs are met. In our platform, this was realized through a flexible consent model that enables digital consent, withdrawal, and revision of consent in response to new data requests. Past research is supportive of dynamic consent as a means to bypass the need for broad consent, as well as a means to enable greater participant autonomy [4,35]. Perhaps one of the most important motivating factors identified in stage 1 for participants regarding engagement and registration was that they received feedback on the use of their data. Underpinning this is the importance of regular communication, which forms the foundation of a trust-based partnership between participants and researchers and has historically been difficult with paper-based consent. Meeting this requirement is made possible through the dynamic consent model, which allows for flexible and minimally invasive communication [4,15-17]. Additionally, participants in stage 1 expressed strong preferences regarding the frequency of contact with researchers, and as such, it is evident that a tailored approach to communication is necessary and that this is an important factor in determining their likelihood to engage with the program.

Contemporary studies regarding dynamic consent support the notion that the model is best realized with a patient-centric design process [36,37]. The Cooperative Health Research in South Tyrol (CHRIS) study used a dynamic consent platform to opportunistically recruit through advertising a closed cohort of 13,000 participants. In contrast, the ATHENA program concept requires ongoing, at-scale, systematic recruitment, which requires a predominantly self-intuitive engagement process [16,23,38]. Users will be able to enter the registration process through means, such as hyperlinks and QR codes. While not fully realized yet, this differs from the CHRIS study, which required the invitation of participants to a study center and close guidance by research assistants present throughout the enrollment process [16]. Moreover, unlike the ATHENA dynamic consent platform, withdrawal from the CHRIS study was only available by contacting the study center. Other examples of dynamic consent platforms, such as that of the Australian Diabetes Data Network (ADDN), utilize recruitment systems that are opportunistic in nature, requiring clinicians to identify relevant patients and then explain the concept of the platform, in order to recruit new participants. This may hinder the recruitment process as it requires significant external input in prompting and registering patients [36].

Our report also provides information on the accessibility and usability of the dynamic consent platform, derived from surveys of users logging in and trialing the platform themselves. In our study, 68% of participants who gave consent to be recontacted were successfully contacted, which is similar to our previously reported recontact success rates using e-consent [23]. A larger proportion of participants in our study logged on to the platform compared to other groups, such as Control (CTRL), who reported <20% dynamic consent platform registration rates [39]. The reasons for this are not clear, but possible reasons are as follows. In CTRL, the “retrospective cohort” was recontacted via email, whereas in our study, patients were telephoned, and this may have affected consent rates. In our study, all patients were preregistered on the consent platform, whereas in CTRL, patients had to register themselves. Finally, in CTRL, the cohort had genetic disorders and consent was required for genetic research, which is a more complex process involving watching a video and answering 13 mandatory and 21 optional consent choices. In our study, the cohort consisted of patients who had COVID-19 previously, and there were only 2 consent choices with an optional survey.

This adds to the small but growing pool of literature on the utility of dynamic consent [38,39]. Notably, the ATHENA dynamic consent platform differs from other studies in that it requests access to all health information of a person, including that from primary care. It also does not focus on any specific group or category of disease, and instead aims to indiscriminately recruit as much of the population as possible over an indefinite period of time. This contrasts with other studies that target specific groups of patients or recruit over specific time periods, such as the Rare and Undiagnosed Diseases Study, ADDN (patients with type 1 diabetes mellitus), CHRIS (genomic research), and CTRL (genomic research) [4,16,17,36].

### Implications for Policy and Practice

The ATHENA program is now ready for implementation in hospitals and health services in Queensland. Previous pilot studies and feasibility assessments, including the ATHENA COVID-19 study, have demonstrated both its operational capabilities and initial effectiveness within smaller contexts [22,23]. Complementing this, an independent economic report on the ATHENA program has confirmed the program’s financial sustainability in terms of return on investment. The next phase involves refining the dynamic consent platform, incorporating insights from this report, and introducing enhanced user authentication mechanisms. These include self-registration, robust user verification utilizing government-issued identification, and 2-factor authentication, ensuring secure access. Another development goal is implementing a posttrial feedback mechanism, empowering participants with insights into the utilization of their data. Additionally, incorporating QR code–based access and registration is necessary to facilitate mass consent. Our study required an initial telephone step in which patients previously registered with the ATHENA COVID-19 study were recontacted to ask if they were interested in receiving information about a new research study and then emailed a login link and password. In the proposed ATHENA program, unregistered patients attending hospital clinics, wards, and procedural areas will be presented with access to the ATHENA website and invited to log in and consent. The authors believe a certain level of human assistance will always be required at certain contact points when using dynamic consent to improve patient experience and ensure maximal recruitment. In the ATHENA program, although human contact will be kept to a minimum, hospital staff will be trained to prompt and invite patients to access the website. Those patients who wish to participate but are unable or unwilling to use dynamic consent will be offered paper-based consent. Staff will also have a level of training so they can explain the ATHENA concept and advise on website access. Furthermore, any patients requiring more information will be invited to telephone the ATHENA information center serviced by ATHENA staff.

### Limitations

This study required patients to be telephoned and provided with the platform weblink and login details, which does not specifically replicate the future intended user experience in a hospital setting. The study also involved a relatively small sample size, which may affect generalizability. Extensive consultation was undertaken with Health Consumers Queensland, First Nations, and culturally and linguistically diverse leaders in the community regarding the ATHENA COVID-19 concept, the ATHENA program, its protocols (including the use of electronic consent), and patient information consent forms. However, the development of the dynamic consent platform was more limited in scope and did not include detailed consultation with these priority groups. Consequently, the validity of its use in these populations is limited. Recognizing this, subsequent platform iterations will involve comprehensive consultations with these groups to co-design a platform with maximum utility and to avoid exacerbating existing inequities in these populations.

### Conclusions

This study outlines the development of the ATHENA dynamic consent platform within the framework of the ATHENA program. It describes the evolution of the platform, its utilization by participants, and the insights gained from this process. The findings showed positive reception, hinging on participant data control, research for the benefit of population health, feedback mechanisms, and protection of data privacy. The platform was well-accepted, highlighting its advantages over traditional methods with regard to flexibility, ease of communication, and participant satisfaction. We hope that this information is useful to other groups who plan to develop a dynamic consent platform for use in health care research.

## Appendix

Multimedia Appendix 1 Sample interview guide.

Multimedia Appendix 2 Stage 4 questionnaire and answer key.

Multimedia Appendix 3 CHERRIES (Checklist for Reporting Results of Internet E-Surveys) table.

Multimedia Appendix 4 Architectural core concept requirements for the dynamic consent platform.

Multimedia Appendix 5 Images of the dynamic consent platform web app user experience.

Multimedia Appendix 6 Flowchart of participant recruitment.

## Abbreviations

ADDN: Australian Diabetes Data Network
ATHENA: Australians Together Health Initiative
CHRIS: Cooperative Health Research in South Tyrol
CTRL: Control

## Data Availability

The datasets used and analyzed in this study are available from the corresponding author upon reasonable request. 
