# Supplementary material for: Insights From the Development of a Dynamic Consent Platform for the Australians Together Health Initiative (ATHENA) Program: Interview and Survey Study
Source: JMIR Form Res. 2024 Nov 6;8:e57165. doi: 10.2196/57165 (PMC11579620; doi:10.2196/57165)
Supplement: Multimedia Appendix 1 [file formative_v8i1e57165_app1.docx]

**Multimedia Appendix 1.** Sample interview guide.

Sample Discussion Guide

1. Interviewer Introduction

- Introduce interviewer name, role.
- Explain the project/program.
- Explain that answers are confidential and completely anonymous.
- Obtain consent to record the interview.
- Answer any questions.

1. Participant Introduction

- Before we get started, can you please tell us a bit about yourself? What do you do, who do you live with?
- We would like to hear about your experiences with technology. Can you tell us a little about how you use technology in your normal day?
- Without revealing anything personal can you please tell us about the last time you went to doctor or a hospital and what type of digital technology you used in your appointment or admission? (e.g., online booking, queue management software, QH website, outpatient portal)

1. Theme 1: Data Knowledge, Trust & Risk

- Can you tell me about the type of data that is kept by your GP or Queensland Health about you or your family?
  - Prompt - medical notes, referrals, Medicare identification.
- How do you feel about your health data being kept by your GP or Queensland Health? Why is that?
  - Prompt - are you happy they have the data or concerned about the data for any reason?
- How does your GP and Queensland Health use the data they have about you or your family?

1. The Concept of Dynamic Consent and Motivations for Sharing Data

- Queensland Health is trying to improve the mental health services that they provide to patients in regional Queensland. By analysing the health data from both GPs and Queensland Health together in relation to people with mental health issues, Queensland Health can then better plan how many mental health wellness clinics and other related services are needed across the State.
  - What is your opinion on sharing this type of data between hospitals and between GPs?
  - Would you like to consent or give permission to this type of data being shared?
  - Would your opinion change if it was also able to help a family member or a good friend?

1. Motivation to sign up and making regular contact.

- Thanks so much for telling us about your experiences. We'd now like to ask you about how much, if and why you would use this future website or online platform.
  - What motivates you to want to sign up to the platform?
  - What barriers do you see would prevent you from signing up to the platform?
  - What type of incentive would increase your desire to sign up? (e.g., charity gets a donation; we pay you money or give you gift vouchers)
  - How many times a week, month or year would you like to be contacted about the platform? Why?
  - How many times a week, month or year would you like to be contacted about the use of your data? Why?
  - Would you like to be contacted about how your data is used?
  - How many times a week, month or year would you like to be contacted about how your data is used?

1. Where would you like to sign up?

- Card sorting task- Place in order from most likely to least likely, the following cards to indicate what would be most influential in you signing up to a dynamic consent solution.
  - Recommended by GP or GP clinic
  - Recommended by healthcare professional in your hospital or other health service
  - Recommended by a charity
  - Recommended by a friend
  - Recommended by a relative
  - Recommended by a government official
  - Recommended by University
  - Other: Explain
- Once completed: please explain the choice of first and last cards.
  - During this, observe what the customer does, what the customer says, and their expression and body language.
  - Would you like to be contacted about participating in this project in the future?
  - Thank participant for their time and answers.
