# Supplementary material for: Insights From the Development of a Dynamic Consent Platform for the Australians Together Health Initiative (ATHENA) Program: Interview and Survey Study
Source: JMIR Form Res. 2024 Nov 6;8:e57165. doi: 10.2196/57165 (PMC11579620; doi:10.2196/57165)
Supplement: Multimedia Appendix 2 [file formative_v8i1e57165_app2.docx]

**Multimedia Appendix 2.** Stage 4 questionnaire and answer key.

| **Question** | **Response score** | | | | |
| --- | --- | --- | --- | --- | --- |
|  | **1** | **2** | **3** | **4** | **5** |
| My overall experience using this website was good | Strongly agree | Agree | Neither agree nor disagree | Disagree | Strongly disagree |
| It was easy to navigate the website | Strongly agree | Agree | Neither agree nor disagree | Disagree | Strongly disagree |
| The length of time it took me to complete my consent choice was too long | Strongly disagree | Disagree | Neither agree nor disagree | Agree | Strongly Agree |
| The information presented to me in the consent choice process was clear | Strongly agree | Agree | Neither agree nor disagree | Disagree | Strongly disagree |
| The amount of information provided to allow me to make a consent choice was just right | Strongly agree | Agree | Neither agree nor disagree | Disagree | Strongly disagree |
| I felt pressured to complete the consent process | Strongly disagree | Disagree | Neither agree nor disagree | Agree | Strongly Agree |
| I liked the opportunity to be able to provide consent choices | Strongly agree | Agree | Neither agree nor disagree | Disagree | Strongly disagree |
| The number of consent choices provided to me were just right | Strongly agree | Agree | Neither agree nor disagree | Disagree | Strongly disagree |
| I felt positively about the opportunity to ask questions and provide feedback to the research team | Strongly agree | Agree | Neither agree nor disagree | Disagree | Strongly disagree |
