# Supplementary material for: Insights From the Development of a Dynamic Consent Platform for the Australians Together Health Initiative (ATHENA) Program: Interview and Survey Study
Source: JMIR Form Res. 2024 Nov 6;8:e57165. doi: 10.2196/57165 (PMC11579620; doi:10.2196/57165)
Supplement: Multimedia Appendix 3 [file formative_v8i1e57165_app3.pdf]

CHERRIES CHECKLIST ATHENA CV-19

**Checklist item**	**Evaluation/response**
Survey Design	Target population were users trialling a dynamic consent platform. The users were randomly selected from a group of participants in a prior study who had consented for re-contact.
Institutional Review Board approval	Ethics approval for this study was granted by the Gold Coast Hospital and Health Service Human Research Ethics Committee (reference HREC/2020/QGC/63555); and the Australian National University Human Research Ethics Committee (reference: 2020/312).
Informed consent	Participants were aware of the investigator, purpose, and length of time of the study.
Data protection	No personal information was collected through our survey.
Development and testing	The survey was developed through collective workshops and modification of a published survey [26]. The technical functionality of the questionnaire was tested through a trial run of the dynamic consent platform prior to deployment.
Open survey vs closed survey	Closed Survey- only participants trialling the dynamic consent platform had access, after utilising the platform to express interest in a clinical trial.
Contact mode	Initial dynamic consent platform users were randomly contacted via telephone from an existing pool of potential volunteers.
Advertising the survey	Registration for the dynamic consent platform (and therefore for the survey) was not advertised but chosen from an existing pool of participants in a previous study who had agreed for re-contact.
Type of e-survey	Web e-survey.
Context	The survey respondents were a closed group of volunteers trialling a dynamic consent platform. They utilised the platform to consent for their details to be released for a clinical trial. The context of the website is unlikely to influence sample selection or results as only select users are granted access.
Mandatory/voluntary	The survey was voluntary.
Incentives	No incentives were offered.
Time/date	The data was collected over a period of two months.
Randomisation of items or questionnaires	The questionnaire items were not randomised or alternated
Adaptive questioning	Adaptive questioning was not utilised
Number of items	One question was displayed per page, each with five Likert scale options.
Number of screens (pages)	The questions were all displayed on 1 page
Completeness check	Participants were unable to submit the questionnaire unless every question had an option selected, and incomplete questions would be highlighted. This was done using JavaScript.
Review step	Participants were able to review and change their answers through user of a ‘back’ button

Unique site visitor	Unique site visitors were not measured however survey responses were linked to individual, unique user logins. Each user account was only allowed to complete the survey once.
View rate	View rates were not measured.
Participation rate	Participation rate was 68% for participants who had completed the consent process.
Completion rate	Completion rate was 100%.
Cookies used	Cookies were not used as survey responses were linked to individual user logins, and each user account was only allowed to complete the survey once.
IP Check	There was no IP check performed as survey responses were linked to individual user logins. After consent, the user was immediately offered a ‘complete the survey’ link.
Log file analysis	Not utilised
Registration	Users only had access to the survey once, which was following consent for the clinical trial. The survey was not displayed thereafter preventing duplicate responses from the same login.
Handling of incomplete questionnaires	Only complete questionnaires were analysed. There were no incomplete questionnaires received.
Questionnaires submitted with an atypical timestamp	There was no timeframe specified for survey completion.
Statistical correction	No methods were applied for statistical correction.
