# Supplementary material for: Insights From the Development of a Dynamic Consent Platform for the Australians Together Health Initiative (ATHENA) Program: Interview and Survey Study
Source: JMIR Form Res. 2024 Nov 6;8:e57165. doi: 10.2196/57165 (PMC11579620; doi:10.2196/57165)
Supplement: Multimedia Appendix 4 [file formative_v8i1e57165_app4.docx]

**Multimedia Appendix 4.** Architectural core concept requirements for the dynamic consent platform.

| **Section** | **Core requirement** |
| --- | --- |
| Platform Business functions | Able to:   - Register, identify and manage consumer details. - Record and manage health consumer consent from multiple sources e.g., importing consent records. - Match and link health consumer identity and their healthcare data for the purpose of linking primary and secondary data. - Obtain primary healthcare data from general practices through automated procedures. - Provide information to health consumers through means of informational videos and web pages. - Integrate data and provide complex data analytics. - Determine candidacy of health consumers to participate in research such that only relevant trials are presented. - Identify and record medical research studies and/or clinical trials using the healthcare or health consumer data provided by ATHENA. - Manage requests for and provision of identifiable and de-identified data for the purposes of medical research studies and clinical trials. |
| Front end user capacity | - Simple and intuitive appearance, interface, and website operation. - Web application which is accessible from tablet, phone, or desktop computer. - Accessible with username and either a digital authentication token or password. - Static pages to be made available:   - Landing page with introductory video   - Website feedback page   - ATHENA Program Research information   - ATHENA Program project information   - ‘About us’ and ‘Contact details’ sections   - Useful links   - Privacy information and policies     - To describe security measures and assurances     - Explanation of how data on the platform will be collected, used, shared, or transferred.     - What information may be stored.     - What types of data are collected. - Interactive pages   - Dashboard displaying new projects available to participant.   - Personal participation page – research projects the participant has already expressed an interest in and consented for their contact details to be released.   - Interactive consent page (customisable consent options for a project) - Interactive functions   - Ability to consent to studies via electronic signature.   - Ability to withdraw consent at anytime |
| Back-end user capacity | - Ability to load consented patient details. - Load additional projects i.e., new clinical trials - Ability to manage data access and remove requests. - Governance system - Load patient surveys - Platform support - Ability to update project participation. - Monitor dynamic consent platform web traffic |

ATHENA: Australians Together Health Initiative.
