# Supplementary material for: Insights From the Development of a Dynamic Consent Platform for the Australians Together Health Initiative (ATHENA) Program: Interview and Survey Study
Source: JMIR Form Res. 2024 Nov 6;8:e57165. doi: 10.2196/57165 (PMC11579620; doi:10.2196/57165)
Supplement: Multimedia Appendix 5 [file formative_v8i1e57165_app5.docx]

**Multimedia Appendix 5.** Images of the dynamic consent platform web app user experience.


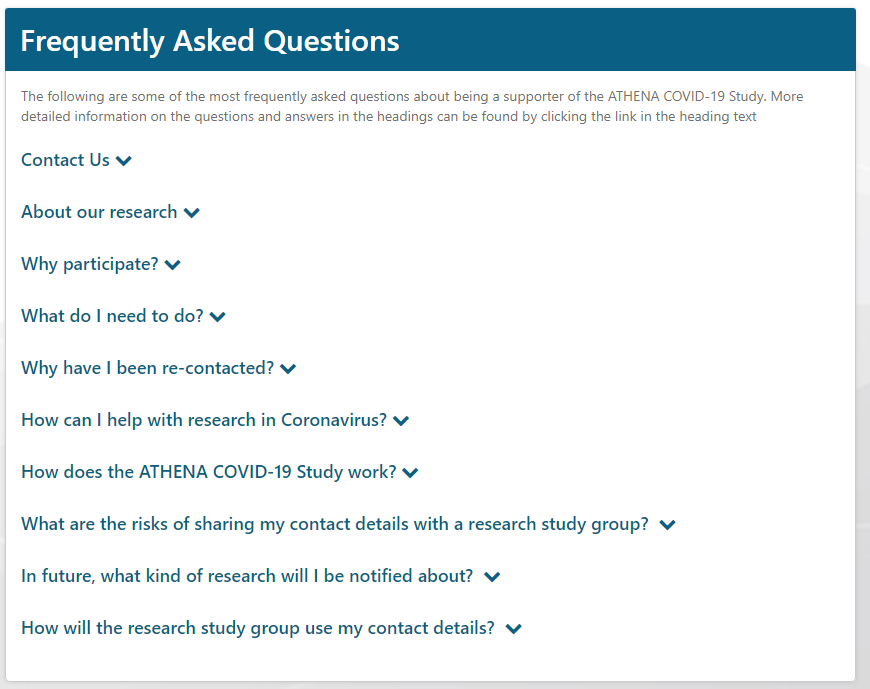


Image A- Frequently Asked Questions


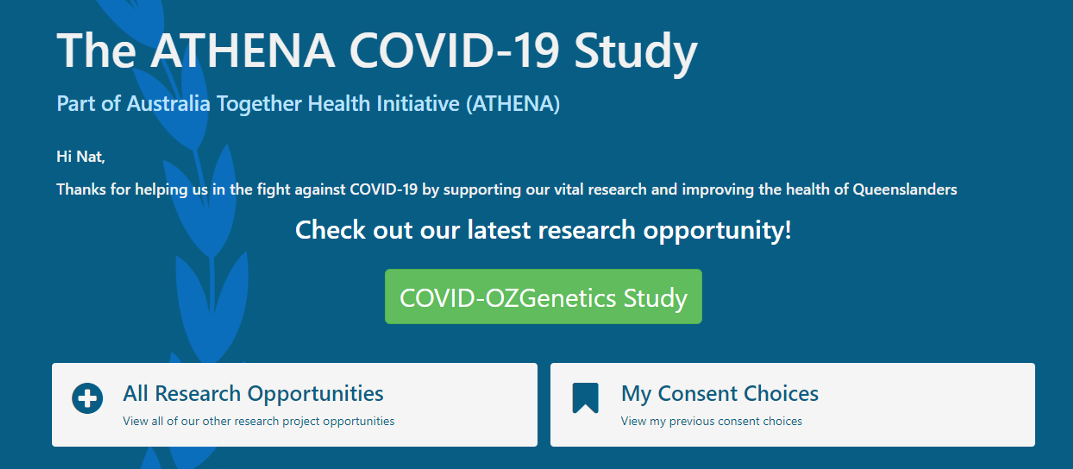


Image B- DCP Landing Page


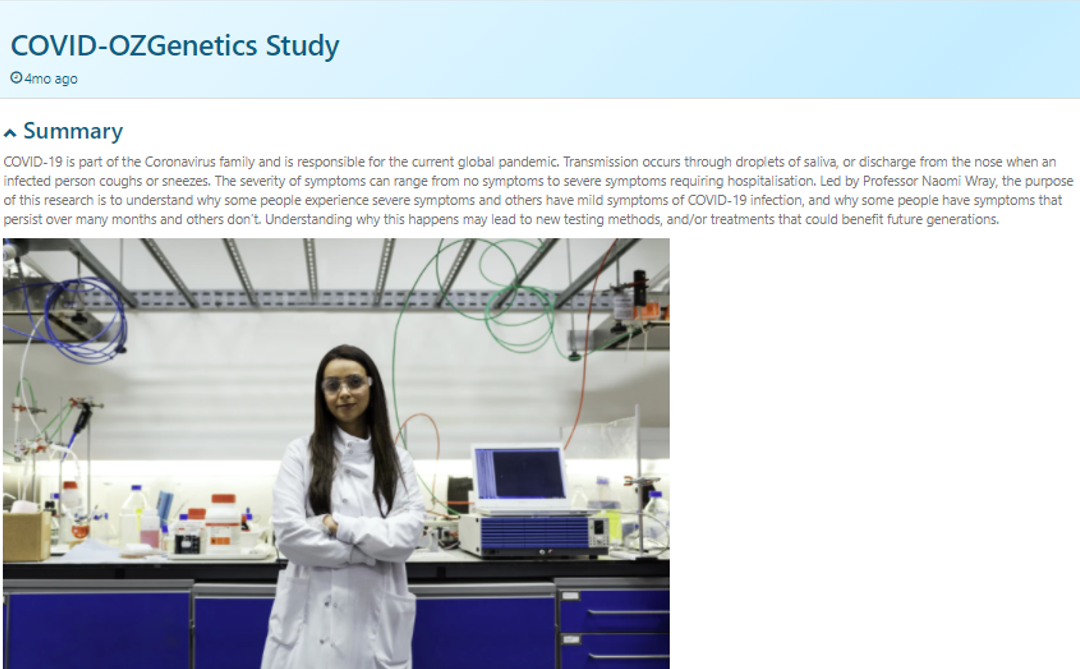


Image C- Sample study display page


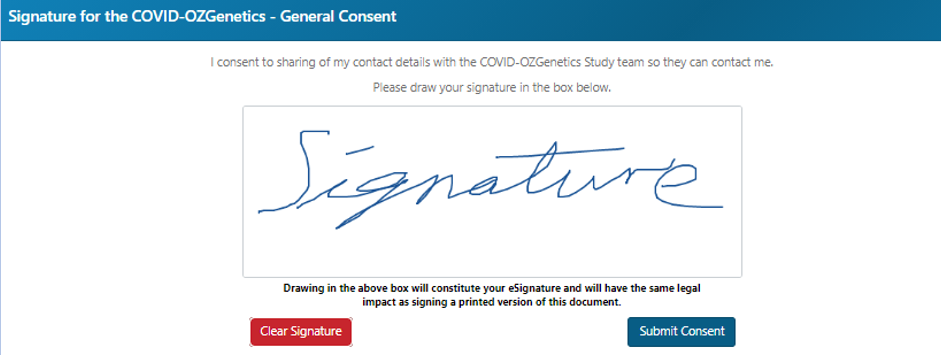


Image D- Digital signature consent form


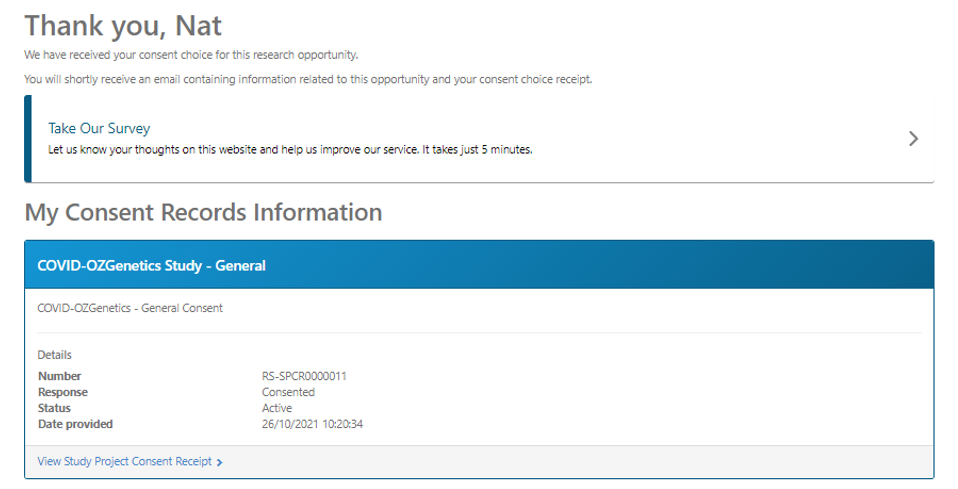


Image E- Digital receipt for consent


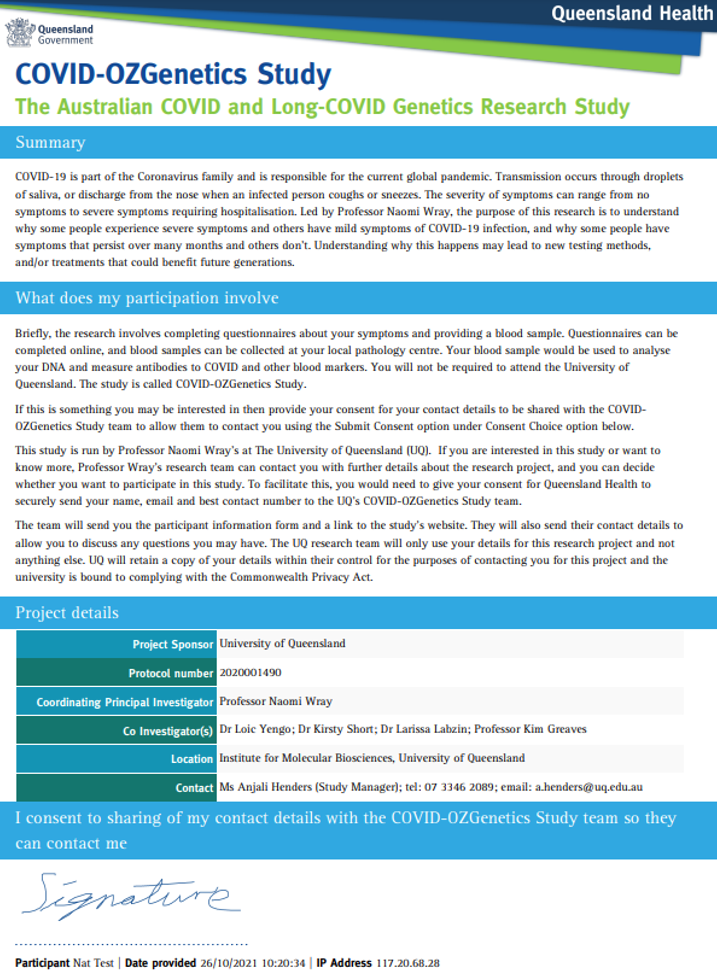


Image F- Confirmation email for submitted consent


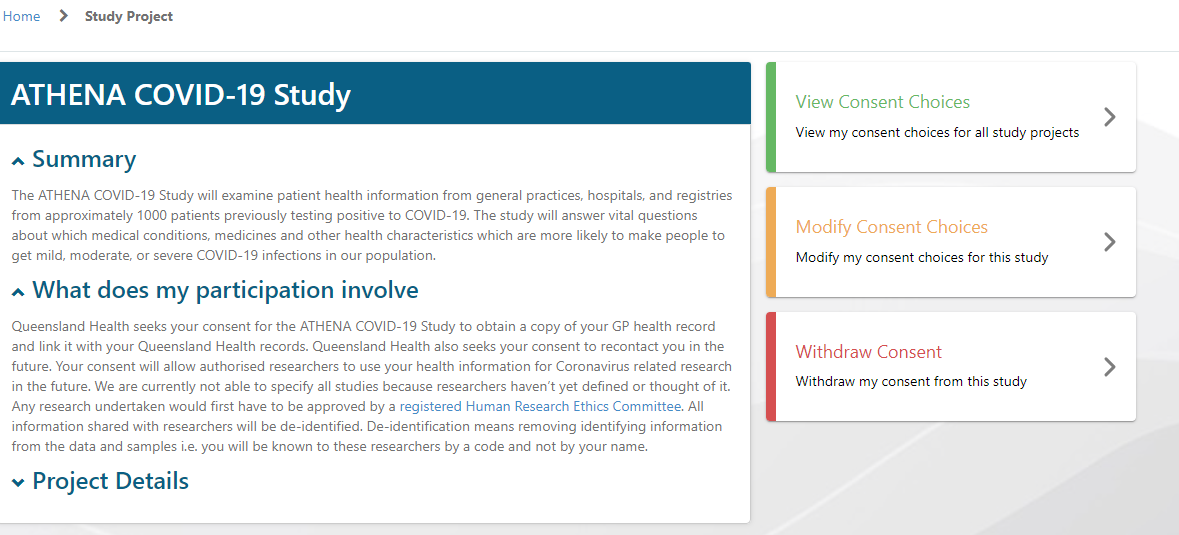


Image G- Withdrawal of Consent
