# Supplementary material for: Insights From the Development of a Dynamic Consent Platform for the Australians Together Health Initiative (ATHENA) Program: Interview and Survey Study
Source: JMIR Form Res. 2024 Nov 6;8:e57165. doi: 10.2196/57165 (PMC11579620; doi:10.2196/57165)
Supplement: Multimedia Appendix 6 [file formative_v8i1e57165_app6.docx]

**Multimedia Appendix 6.** Flowchart of participant recruitment.


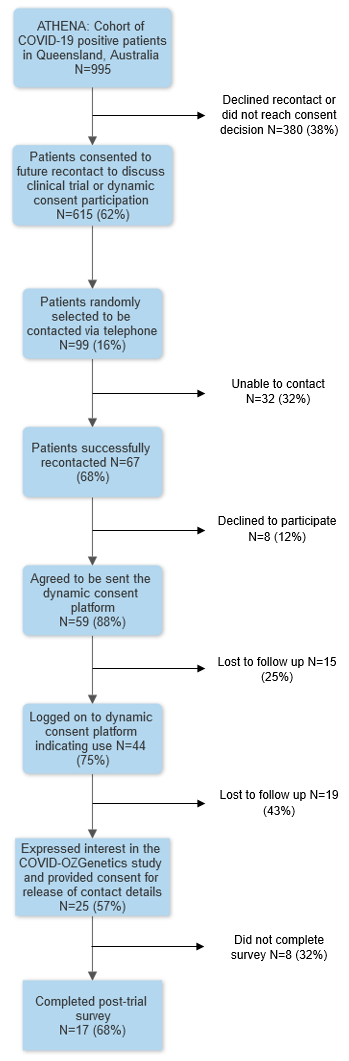


ATHENA: Australians Together Health Initiative. COVID-19: Coronavirus Disease.
